# Virtual Screening Identifies Chebulagic Acid as an Inhibitor of the M2(S31N) Viral Ion Channel and Influenza A Virus

**DOI:** 10.3390/molecules25122903

**Published:** 2020-06-24

**Authors:** Maggie C. Duncan, Pascal Amoa Onguéné, Ibuki Kihara, Derrick N. Nebangwa, Maya E. Naidu, David E. Williams, Aruna D. Balgi, Kerstin Andrae-Marobela, Michel Roberge, Raymond J. Andersen, Masahiro Niikura, Fidele Ntie-Kang, Ian Tietjen

**Affiliations:** 1 Faculty of Health Sciences, Simon Fraser University, Burnaby, BC V5A 1S6, Canada; maggie_duncan@sfu.ca (M.C.D.); ikihara@sfu.ca (I.K.); maya_naidu@sfu.ca (M.E.N.); masahiro_niikura@sfu.ca (M.N.); 2 Department of Chemistry, University Institute of Wood Technology Mbalmayo, University of Yaoundé I, Mbalmayo, Cameroon; amoapascal2@gmail.com; 3 Department of Biochemistry and Molecular Biology, University of Buea, CM-00237 Buea, Cameroon; nebaderrick@gmail.com; 4 Departments of Chemistry and Earth, Ocean & Atmospheric Sciences, University of British Columbia, Vancouver, BC V6T 1Z4, Canada; davewill@chem.ubc.ca (D.E.W.); raymond.andersen@ubc.ca (R.J.A.); 5 Department of Biochemistry and Molecular Biology, University of British Columbia, Vancouver, BC V6T 1Z3, Canada; balgi@mail.ubc.ca (A.D.B.); michelr@mail.ubc.ca (M.R.); 6 Department of Biological Sciences, University of Botswana, Gaborone, Botswana; marobelak@mopipi.ub.bw; 7 Department of Chemistry, University of Buea, CM-00237 Buea, Cameroon; 8 Institute of Pharmacy, Martin-Luther University Halle-Wittenberg, 06120 Halle, Germany; 9 Institute of Botany, Technical University of Dresden, 01217 Dresden, Germany; 10 The Wistar Institute, Philadelphia, PA 19104, USA

**Keywords:** influenza A virus, chebulagic acid, M2, antivirals, natural products, viroporin

## Abstract

The increasing prevalence of drug-resistant influenza viruses emphasizes the need for new antiviral countermeasures. The M2 protein of influenza A is a proton-gated, proton-selective ion channel, which is essential for influenza replication and an established antiviral target. However, all currently circulating influenza A virus strains are now resistant to licensed M2-targeting adamantane drugs, primarily due to the widespread prevalence of an M2 variant encoding a serine to asparagine 31 mutation (S31N). To identify new chemical leads that may target M2(S31N), we performed a virtual screen of molecules from two natural product libraries and identified chebulagic acid as a candidate M2(S31N) inhibitor and influenza antiviral. Chebulagic acid selectively restores growth of M2(S31N)-expressing yeast. Molecular modeling also suggests that chebulagic acid hydrolysis fragments preferentially interact with the highly-conserved histidine residue within the pore of M2(S31N) but not adamantane-sensitive M2(S31). In contrast, chebulagic acid inhibits in vitro influenza A replication regardless of M2 sequence, suggesting that it also acts on other influenza targets. Taken together, results implicate chebulagic acid and/or its hydrolysis fragments as new chemical leads for M2(S31N) and influenza-directed antiviral development.

## 1. Introduction

Influenza A virus is responsible for recurring seasonal and pandemic outbreaks that cause significant morbidity and mortality worldwide. While, vaccination remains the most effective method of influenza prevention and control, it is not always effective against emerging and circulating seasonal influenza strains, and pandemic strains can spread faster than the development of an effective vaccine. While several classes of influenza antivirals are available and widely used, the risk of resistance to these antivirals continues to be a concern [1]. Therefore, the discovery and development of new antivirals against influenza A remain needed.

**Figure 1:**
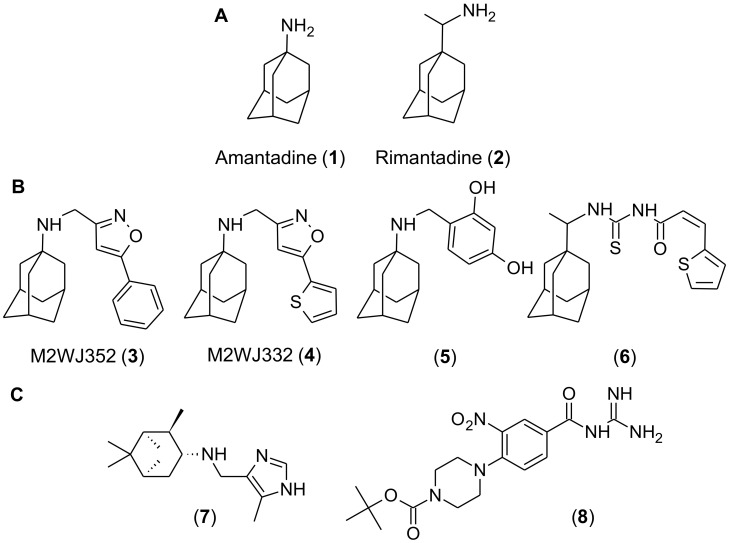
(**A**) Chemical structures of amantadine and rimantadine. (**B**,**C**) M2(S31N) inhibitors assembled for VS and determination of shared pharmacophores. Inhibitors were grouped into adamantine (**B**) and non-adamantane (**C**)-class inhibitors.

M2 of influenza A virus is a 97 amino acid, type I transmembrane protein that forms a tetrameric ion channel that is proton-gated and proton-selective [2,3,4]. Following viral entry, M2 channels, expressed on the virion surface conduct protons, from increasingly acidic host cell endosomes into the virion interior, thereby promoting viral-host membrane fusion and viral ribonucleoprotein release into the host cell cytoplasm. In some cases, M2 on host cell membranes can also conduct protons to elevate secretory vesicle pH, which in turn, delays egress of nascent viral particles and prevents viral hemagglutinin from prematurely adopting a nonfunctional, low pH conformation [5,6]. M2 ion channel activity is essential for influenza A virus replication, and the licensed M2 inhibitors amantadine (1) and rimantadine (2; Figure 1A), which target and occlude the M2 channel pore, were historically used as antivirals. However, drug-resistant virus strains are now so prevalent that adamantanes are no longer recommended for use [1,7,8]. More than 95% of adamantane-resistant influenza A virus strains contain a serine to asparagine mutation at position 31 in M2 (S31N) [7]; this mutation distorts interactions of adamantanes with M2 pore-lining residues without affecting M2 ion channel activity or viral fitness [4]. However, while several adamantane- and non-adamantane-based inhibitors of M2(S31N) have been recently described [4], none are currently licensed for use in humans. Therefore, the discovery of additional inhibitors of M2(S31N), including those representing distinct and, as of yet, unconsidered chemical scaffolds, is warranted as part of ongoing efforts to restore this therapeutic gap.

One method toward identifying new antivirals involves virtual screening (VS) of compound databases followed by functional validation of screening hits and molecular docking studies [9,10,11,12]. In this approach, one or more pharmacophores shared by bioactive compounds are first identified. These pharmacophores are then used to probe virtual chemical libraries for related structural configurations. We previously reported the use of VS to identify novel inhibitors of HIV-1 replication and HIV latency reversal, thereby demonstrating the feasibility of this approach [13,14]. We and others have also shown that libraries of pure compounds, derived from natural products, are rich sources of antivirals with distinct structural diversity [13,15,16,17,18].

Based on these observations, we hypothesized that screening of natural product-derived chemical libraries may identify new M2(S31N) inhibitors and influenza antivirals. We, therefore, performed a VS-based study of pure compound libraries derived from marine and terrestrial sources in addition to the pan-African Natural Products Library (p-ANAPL) [13,19]. We report here the results of this screen and discovery of chebulagic acid as an inhibitor of influenza replication, which also acts on M2(S31N).

## 2. Results

### 2.1. Discovery of Chebulagic Acid as a Candidate M2(S31N)Inhibitor by Virtual Screening

To identify shared pharmacophores that may underlie M2(S31N) inhibition, we assembled a series of adamantane and non-adamantane-based compounds that were previously reported to inhibit M2(S31N) bioactivities (Figure 1B,C). For adamantane-based structures, four compounds were selected (Figure 1B). These included M2WJ352 and M2WJ332 (compounds **3** and **4**, respectively), which are reported to selectively inhibit M2(S31N), as measured by two-electrode voltage clamp (TEVC)-based electrophysiology (reported half-maximal inhibitor concentration (IC_50_) of M2 current = 14, and 16 µM, respectively). In contrast, neither blocked more than 20% of M2(S31) currents at 100 µM, indicating selectivity for M2(S31N). Compounds **3** and **4** were also reported to inhibit replication of influenza A virus with M2(S31N), as measured by plaque reduction assay (reported half-maximal effective concentration (EC_50_) = 14 and 0.1 µM, respectively) [20]. We also selected compound **5**, a benzodiol derivative, which was reported to block both M2(S31) and M2(S31N) currents in TEVC (IC_50_s = 60 and 35 µM, respectively) and viral replication in plaque reduction assays (EC_50_s = 1 and 3.2 µM) [21]. Although not assessed by TEVC or plaque reduction assay, a thiophene derivative (compound **6**) was selected as it restores growth of bacteria expressing M2(S31N), which would otherwise inhibit bacterial growth, with an EC_50_ of 25 µM [22,23]. We also selected two non-adamantane based compounds with reported activity against M2(S31N) (Figure 1C). Compound **7**, a pinanamine derivative, was reported to inhibit 27% of M2(S31N) and 96% of M2(S31)-dependent currents at 100 µM, as measured by TEVC, although it was effective only against M2(S31)-containing viruses in plaque reduction assays [24]. In contrast, compound **8**, a derivative of hexamethylene amiloride, inhibited 32% of M2(S31N)-dependent currents at 100 µM, but not M2(S31) currents, as measured by single electrode voltage clamp electrophysiology of M2-transfected HEK cells [25].

**Figure 2:**
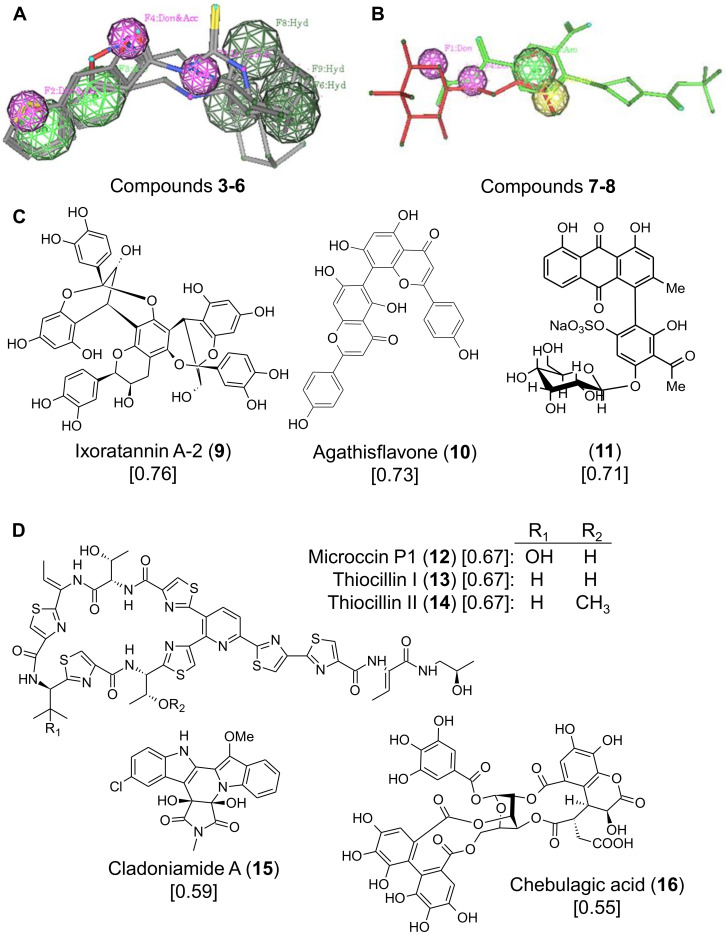
(**A**,**B**) Alignment of adamantane-class inhibitors (**A**) and non-adamantane-class inhibitors (**B**). Chemical moieties that define shared pharmacophores are highlighted. (**C**) Structures of three p-ANAPL molecules identified with pharmacophores shown in **A**,**B**. (**D**) Structures of five marine and terrestrial natural product molecules with shared pharmacophores. For each compound, the identified RMSD value is shown.

To identify common pharmacophores for these compounds, three-dimensional conformations were generated using the (default) MMFF94x force field [26], which was implemented using Molecular Operating Environment (MOE) software. To accomplish this, we used the protocol of Daveu et al. (1999) [27], as we were previously successful with this method to identify inhibitors of HIV replication by VS from natural product libraries [13]. The six selected M2(S31N) inhibitors, used to identify potentially bioactive pharmacophores, were split in two based on the presence (compounds **3** to **6**) or absence (compounds **7** and **8**) of the adamantane ring system. As a result, two sets of pharmacophore queries were generated; one from analogues containing the adamantane group (Figure 2A); the other from the remaining compounds (Figure 2B). The common pharmacophore features derived from the superposition of compounds **3** to **6** (pharmacophore I) included the hydrophobic centers around the adamantane moiety (Hyd), the donor/acceptor features on the NH group of the secondary amine (Don&Acc), and the hydrophobic center represented by the benzene ring in compound **3** and the thiophene in compounds **4** and **6** (Hyd) (Figure 2A). In contrast, the common pharmacophore features from superposing compounds **7** and **8** (pharmacophore II) included the aromatic feature of the benzene ring in compound **8** (shown in green on Figure 2B), which coincided with the aromatic feature (Aro) of the imidazole ring of compound **7** (shown in red on Figure 2B), in addition to one of the acceptor features of the piperazine in compound **8** superposing upon the donor-acceptor feature of the NH group of the secondary amine in compound **7** (Figure 2B).

The common pharmacophore features of the six compounds described in Figure 2A,B were then used in the virtual screening of the previously virtualized p-ANAPL and the newly virtualized marine and terrestrial natural products compound library (See Materials and Methods) [18,19]. We chose natural product libraries for searching by VS, based on our previous observations that the chemical diversity of these libraries [18,19] may increase the likelihood of identifying bioactive viral inhibitors [13,17]. Using pharmacophore I, a “hit” list comprising 6 compounds from the marine and terrestrial natural product library and 10 from p-ANAPL were identified with the lowest RMSD values ranging from 0.87 to 0.99 and from 0.76, to 0.95, respectively. Using pharmacophore II identified 7, and 22 compounds, respectively, with the lowest RMSD values ranging from 0.49 to 0.67 and 0.45 to 0.75. These compounds were prioritized for “drug-likeness” including no more than 2 Lipinski violations, the number of pharmacophore features of the hit compound, and the feasibility for further medicinal chemistry oriented synthesis (often indicated by higher computed synthetic accessibility scores), as previously described [19]. We also prioritized compounds by how many milligrams of the compound sample were available for in vitro validation. As a result, 8 compounds (**9–16**) were selected for further biological validation and included 3 from p-ANAPL (Figure 2C) and 5 from the marine and terrestrial natural product library (Figure 2D). The latter set included chebulagic acid (compound **16**), which also had the lowest RMSD value of selected compounds obtained by the VS (0.55 against pharmacophore II).

### 2.2. Chebulagic Acid Inhibits M2(S31N)Activity In Vitro

To assess the ability of VS hits to inhibit M2 activity in vitro, we employed a previously-described yeast growth restoration assay [28,29]. Briefly, *Saccharomyces cerevisiae* strains contain a multicopy plasmid of M2(S31N) or M2(S31) from the Udorn strain of influenza A under the control of the inducible GAL1 promoter. As a result, galactose-induced M2 expression inhibits yeast growth over time, as measured by culture turbidity. However, the co-incubation of galactose-treated cells with non-toxic inhibitors of M2 restores yeast growth.

**Figure 3:**
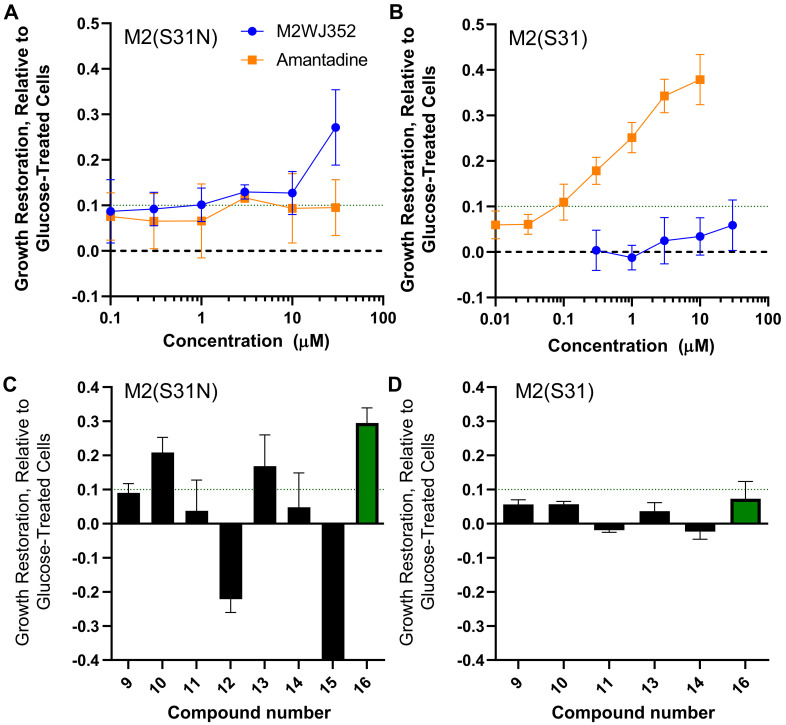
Ability of compounds to restore growth in yeast expressing M2. (**A**,**B**) Restoration of yeast growth in M2(S31N) (**A**) and M2(S31)-expressing cells (**B**) in the presence of the control M2(S31N) inhibitor M2WJ352 and control M2(S31) inhibitor amantadine. (**C**,**D**) restoration of yeast growth in M2(S31N) (**C**) and M2(S31)-expressing cells (**D**) in the presence of natural products shown in Figure 2 (compounds **9**–**16**). Effects of chebulagic acid are highlighted in green.

To validate the use of this assay, we induced expression of M2(S31N) in yeast in the presence of the control M2(S31N) inhibitor M2WJ352 (compound **3**) or control M2(S31) inhibitor amantadine (**1**). After 20 hours’ incubation, expression of M2(S31N) reduced yeast growth to 30.6 ± 11.6% (mean ± SD) of the strain treated with glucose, while expression of M2(S31) reduced growth to 24.4 ± 12.9% of glucose-treated cells. However, while the growth of the M2(S31N)-expressing yeast strain was not affected by the addition of up to 30 µM amantadine (i.e., restoring < 10% of yeast growth), incubation with 30 µM M2WJ352 induced an average of 27.1 ± 8.3% increased growth relative to untreated M2(S31N)-expressing cells (Figure 3A), consistent with the selective inhibition of M2(S31N) by M2WJ352 but not amantadine. Conversely, while M2WJ352 did not substantially restore growth of M2(S31N)-expressing yeast at up to 30 µM, amantadine restored growth with clear dose-dependence. For example, 0.3 µM amantadine restored an average of 17.8 ± 3.0% yeast growth in 3 independent experiments, while 10 µM restored up to 37.9 ± 5.6% growth (Figure 3B). These results are consistent with previously reported growth restoration data [29] and the inhibitory properties of amantadine and M2WJ352 as measured by electrophysiology [20,25].

We next assessed the ability of the 8 compounds identified from the VS to restore growth of M2(S31N)-expressing yeast at 25 µg/mL (Figure 3C). Two compounds (**12** and **15**) resulted in substantially reduced turbidity (22.1 ± 3.9 and 70.4 ± 0.1% reduced growth, respectively) and clear cell death as observed by microscopy and were not considered further. However, three compounds restored at least 10% yeast growth at 25 µg/mL including compounds **10**/agathisflavone (20.9 ± 4.4%), **13**/thiocillin I (16.9 ± 9.2%), and **16**/chebulagic acid (29.5 ± 4.4%) (Figure 3C). These results suggest that a subset of compounds identified by VS might counteract the detrimental effects of M2(S31N) expression on yeast growth, where the activity of 25 µg/mL (~26.2 µM) chebulagic acid is on par with the activity of 30 µM M2WJ352. Notably, none of the 8 compounds restored > 10% growth of yeast expressing M2(S31), with all observed activities within the biological noise of the assay (Figure 3D). These results suggest that chebulagic acid restores growth in yeast cells selectively expressing M2(S31N).

### 2.3. Molecular Simulation of Chebulagic Acid with Both Wild-Type and Mutant Forms of M2 Viroporin

**Figure 4:**
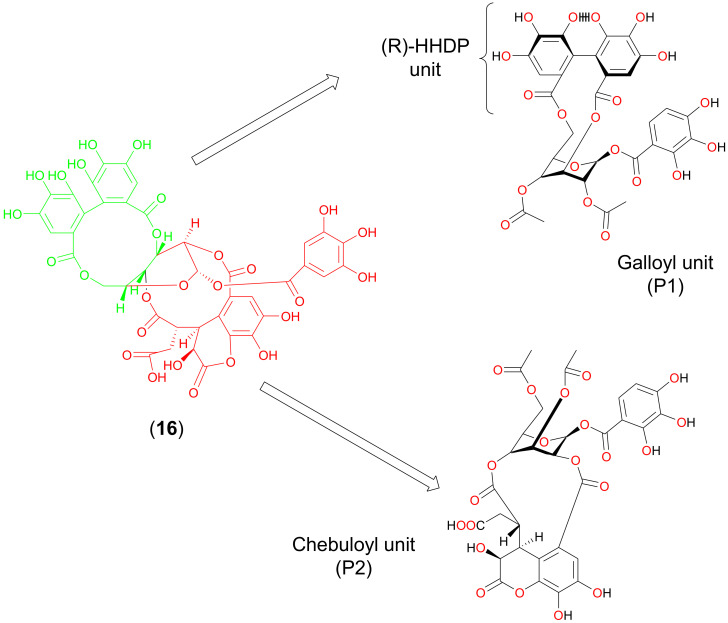
Summary of the expected hydrolysis fragments of chebulagic acid (P1 and P2) used for molecular modelling.

**Figure 5:**
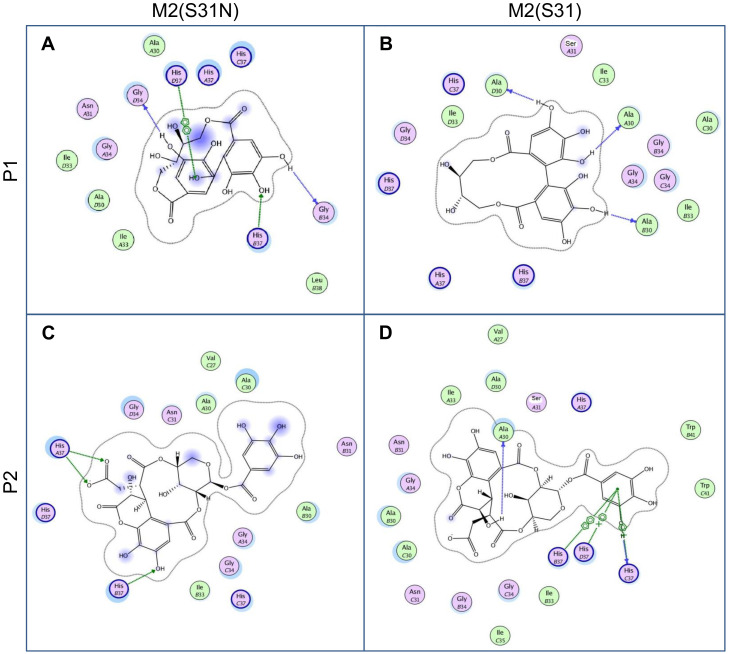
Relative 2D protein-ligand interaction maps of P1 and P2 into M2(S31N) and M2(S31). For each panel, the proposed ligand-protein interactions, amino acids and M2 subunit (**A**−**D**) are described as follows; green arrows are H-bond acceptors, blue arrows are H-bond donors, and green lines are π-π and cation-π interactions.

**Figure 6:**
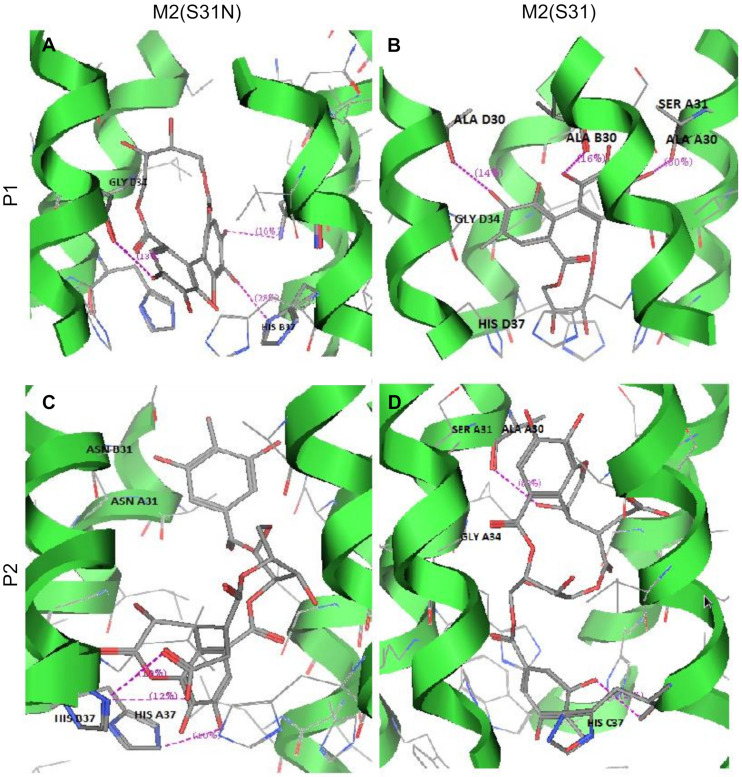
3D pictures of protein-ligand interactions between P1 and P2 into M2(S31N) and M2(S31). For each panel, the proposed ligand-protein interactions, amino acids and M2 subunit (**A**−**D**) are labelled, with H-bonds shown as pink broken lines.

To investigate how chebulagic acid may interact with M2, we next performed molecular docking studies with it and the M2 transmembrane domain tetramer (PDB code: 2LY0, NMR structure of residues 19–49 of M2 (H3N2) in dodecylphosphocholine micelles) [20]. This was computationally modified to include S31 when necessary, as described in the literature [21]. As chebulagic acid was too large to fit within the M2 pore, we assumed that only one or more portions of the molecule were functionally active. We investigated the docking of two predicted hydrolysis reaction products: the galloyl unit (P1) and the chebuloyl unit (P2) (Figure 4). P1 and P2 were separately docked to both M2(S31N) and M2(S31), and the top-ranking poses for each docked complex with the tightest binding affinities were selected and analyzed. Computed binding affinities for P1 and P2 towards M2(S31N) and M2(S31) are shown in Table 1. Protein-ligand interactions for docking poses of P1 and P2 are further shown in 2-dimensional configuration in Figure 5 and in 3 dimensions in Figure 6.

In both cases (docking with P1 and P2), we observed that conformations with lower binding energies were observed when the ligand interacted with the S31N form of M2. In comparison, the docking of P2 with M2(S31) resulted in a positive (i.e., lack of) binding affinity and unfavorable interactions between the docked ligand and the protein (Figure 5D). The enthalpic contributions to the free binding energies of binding (ΔG) obtained from the Affinity dG scoring shown in Table 1 further suggested more favorable binding of P1 and P2 within M2(S31N)(ΔG = −39.75 and −30.17 kcal/mol, respectively), when compared with binding to the S31 form.

The highly conserved histidine residue at position 37 (H37) within the M2 pore is responsible for shuttling protons through the channel [4] and is essential for M2 activity. The docking data suggest that, in the presence of Asn at position 31, a series of stabilizing H-bond interactions are elicited including between H37 and the chebulagic acid hydrolysis fragments (Figure 5A,C; Figure 6A,C). In the context of M2(S31N), these interactions would be predicted to disrupt proton flow across the H37 proton shuttle, thereby inhibiting M2(S31N). In contrast, none of these interactions were observed in the context of M2(S31) for P1 (Figure 5B and Figure 6B), while only weak cation-π interactions were observed for P2 (Figure 5D and Figure 6D). These results suggest that chebulagic acid hydrolysis fragments selectively interact with M2(S31N) and its H37 residue to occlude the M2(S31N) pore.

### 2.4. Chebulagic Acid Inhibits Influenza Virus Replication

To confirm whether the observed selectivity of chebulagic acid to inhibit M2(S31N) extended to antiviral activities, we assessed the ability of chebulagic acid to inhibit influenza A virus replication. We used a previously described reverse genetic system [30] to generate A/Puerto Rico/8/34 (PR8) strains that encode M2 exclusively with N31 or S31 (PR8_M2(S31N)_ or PR8_M2(S31)_, respectively). In both cases, the endogenous T27 mutation was also reverted to the wild-type V27 sequence. Viruses were first used in assays to measure inhibitory concentrations, as described previously [31], where target Madin-Darby canine kidney (MDCK) cells were observed for morphological changes, consistent with virus-mediated toxicity, 2 and 3 days following infection with a 50 * median tissue culture infection dose (TCID_50_) of virus.

Using this approach, amantadine was observed to selectively inhibit cytopathic effects in MDCK monolayers due to PR8_M2(S31)_ (minimal inhibitory concentration = 1.8 ± 2.3 µM) but not PR8_M2(S31N)_ as expected (inhibitory concentration > 100 µM; Table 2). Similarly, M2WJ352 selectively inhibited cytopathic effects in monolayers infected with PR8_M2(S31N)_ (inhibitory concentration = 17.6 ± 19.3 µM; mean ± SD) but not PR8_M2(S31)_ (inhibitory concentration > 100 µM). Notably, in this assay chebulagic acid also inhibited cytopathic effects due to PR8_M2(S31N)_ with an inhibitory concentration of 17.2 ± 15.2 but also inhibited cytopathic effects due to PR8_M2(S31)_ (inhibitory concentration = 32.4 ± 24.7 µM; Table 2). No evidence of cytotoxicity was observed with up to 100 µM chebulagic acid. Further, no antiviral activity was observed for compounds **10** and **13** at up to 75 µg/mL (approximately 140, and 65 µM, respectively).

We then assessed the ability of chebulagic acid to inhibit virus replication in plaque reduction assays as described previously (Table 3) [25,30]. As expected, amantadine effectively inhibited PR8_M2(S31)_ replication (EC_50_ = 0.16 ± 0.02 µM; mean ± s.e.m.) but not PR8_M2(S31N)_ (EC_50_ > 5 µM), while M2WSJ352 selectively inhibited PR8_M2(S31N)_ (EC_50_ = 3.2 ± 1.2 µM) over PR8_M2(S31)_ (EC_50_ = 32.7 ± 16.1 µM). In contrast, chebulagic acid inhibited both PR8_M2(S31N)_ and PR8_M2(S31)_ (EC_50_s = 60.9 ± 22.0 and 50.3 ± 26.4 µM, respectively). Taken together, these results indicate non-selective antiviral activity by chebulagic acid against both M2(S31N) and M2(S31)-containing strains of influenza.

## 3. Discussion

Due to the recurring threats of novel seasonal and pandemic influenza strains, antivirals that can effectively supplement existing vaccination strategies continue to be needed. This is particularly the case against the M2 viral protein, as nearly all currently circulating virus strains are resistant to licensed M2-based antivirals due to N31 in M2. Here we describe a VS-based approach to identify natural product-derived pure compounds which structurally resemble known M2(S31N) inhibitors. In cell culture, chebulagic acid also restores the growth of M2(S31N)-expressing yeast cells and inhibits virus replication. Although it is formally possible that this observation is due to the ability of chebulagic acid to restore yeast growth by a mechanism other than M2, this appears unlikely as growth restoration was observed by 25 µg/mL chebulagic acid only in the presence of M2(S31N) and not M2(S31). We also show that chebulagic acid hydrolysis fragments interact within the M2 pore to block proton transport in molecular modeling studies. Chebulagic acid represents a novel, putative chemical scaffold from which to develop additional inhibitors of adamantane-resistant M2.

Chebulagic acid is a hydrolysable tannin most frequently reported to originate from *Terminalia chebula* Retz. [32], although it has also been isolated from several other plants [33,34,35]. It is reported to exhibit numerous in vitro and/or in vivo bioactivities, including anti-inflammatory [36,37,38], anti-angiogenic [39,40], antitumor [41,42], and glucose uptake regulation properties [43,44,45,46], among others. Chebulagic acid is also reported to exhibit broad-spectrum antiviral activity through interfering with multiple viral targets. For example, it inhibits the entry of herpes simplex virus 1 and other viruses by antagonizing the interactions of viral glycoprotein and cell-surface glycosaminoglycans [47,48,49,50]. It can also inhibit the viral NS3–4A protease of hepatitis C at low micromolar concentrations [33] and may also target the capsid protein of coxsackievirus A16 [51]. Notably, 1 mg/kg chebulagic acid also reduced mouse mortality following a lethal dose of human enterovirus 71, indicating that antiviral activity extends to in vivo models [52].

More recently, chebulagic acid was reported to inhibit in vitro replication of a panel of influenza A viruses including PR8, as determined by viral yield reduction assays where culture supernatants of infected MDCK cells plus inhibitors were harvested at 24 h post-infection and titered against fresh target MDCK cells [53]. In these assays, the authors observed EC_90_s in the single micromolar range (e.g., 1.26 µM), or comparable to the control inhibitor oseltamivir carboxylate (EC_90_ = 3.92 µM), while less activity was observed against influenza B viruses. Interestingly, using a reporter virus with single-cycle infection conditions, the authors also showed no inhibitory effect on influenza A virus entry or RNA replication. Rather, chebulagic acid was observed to block viral release and neuraminidase activity in an enzyme inhibition assay, although the latter activity was observed at 100-fold higher concentrations than what was observed to inhibit viral replication [53].

Consistent with these observations, we also observe that chebulagic acid inhibits viral replication in both 50× TCID_50_ and plaque reduction assays. However, we observed antiviral activities at much higher concentrations (i.e., ~17–34 µM in 50× TCID_50_-based cytopathic assays and notably only ~50–61 µM in plaque reduction assays), which may potentially correspond to M2-independent functions. While, the weaker antiviral activity of chebulagic acid is not immediately clear, these differences could reflect our use of assays that measure primary effects of chebulagic acid on viral replication, as opposed to secondary effects of infected culture supernatants reported previously [53]. We also observed similar inhibition of virus strains containing either M2(S31N) or M2(S31), which further supports that chebulagic acid is likely to act on one or more additional viral or host targets independent of M2.

However, using an established yeast growth restoration assay and molecular modelling, we show that chebulagic acid, or at least its hydrolysis fragments, also interfere with M2(S31N). As M2 is required for the initiation of viral-host membrane fusion and viral ribonucleoprotein release into the cytoplasm [4], these results initially appear at odds with the recent observations from Li et al. (2020) [53]. One possibility is that, while chebulagic acid and/or its hydrolysis fragments are able to inhibit M2 in vitro, this inhibition is insufficient to fully block viral entry into the host cytoplasm. Alternatively, chebulagic acid’s effects on M2 may be more relevant in inhibiting viral egress, where M2 is also reported to play a role [5,6].

Taken together, chebulagic acid is likely to inhibit influenza A virus by targeting multiple mechanisms including M2(S31N) and also serves as a chemical starting point for the development of potential M2(S31N) inhibitors, which are structurally distinct from existing agents. The development of chebulagic acid derivatives as influenza antivirals may also be attractive as its ability to target multiple viral factors may reduce the risk of emerging viral resistance over time. However, further studies are necessary to determine the antiviral potential of chebulagic acid. For example, viral outgrowth studies in the long-term presence of chebulagic acid may help to identify resistance mutations in M2, neuraminidase, and/or other viral proteins. Future studies of the effects of chebulagic acid and/or analogues on M2 should also be performed using electrophysiological measures, such as TEVC, although care must be taken to ensure that sufficient hydrolysis fragments are available to allow for the proposed interactions of chebulagic acid with the M2 pore.

## 4. Materials and Methods

### 4.1. Materials and Reagents

MDCK cells were obtained from the American Tissue Culture Collection (ATCC, Manassas, VA, USA). Cells were cultured in Dulbecco’s MEM (Life Technologies, Burlington, ON, Canada) plus 10% fetal calf serum, 100 U/mL penicillin, and 100 µg/mL streptomycin (DMEM+). All p-ANAPL and marine and terrestrial natural product library compounds were previously confirmed to be at least 95% pure [18,19]. Amantadine hydrochloride was obtained from Sigma, Oakville, ON, Canada. M2WJ352 was synthesized as described previously [20,25]. Additional sources of chebulagic acid were obtained as a powder from MedChemExpress (Monmouth Junction, NJ, USA).

### 4.2. Pharmacophore-Based Virtual Screening Methods

Virtual screening was carried out using the Pharmacophore Query Editor implemented within the Molecular Operating Environment software 2012 version (MOE, Chemical Computing Group, Montreal, Canada). Briefly, during the preliminary molecular modelling, leading to the generation of low energy conformations of the bioactive compounds reported in the literature, the force field parameters were kept at their default values of the strain limit of 4 kcal/mol and conformations limit of 250 conformations/molecule. The other settings were kept at their default values, except for the ‘split output’ option and ‘input filters’ which were turned off. Using the MOE Pharmacophore Query Editor, a pharmacophore query was created that consisted of a set of constraints on the location and type of pharmacophore features to be used for searching the database for molecular conformations. The query was created and saved, to be used later in a pharmacophore search.

The common pharmacophore features of the six compounds described in Figure 1 were then used in the virtual screening of both the previously virtualized p-ANAPL and the newly virtualized marine and terrestrial natural products compound library. Two pharmacophore queries were performed. For query 1, compounds with at least six common query features were selected as potential leads in this search, while for query 2, compounds with at least three common query features were selected.

Selected leads were measured against a database that was already enriched by a factor of 10 using MACCS fingerprints, while features for an aligned set of compounds were suggested by using a pharmacophore consensus. The implemented algorithm then generates very similar compounds (10 times the number of known input active compounds) to prepare an initial screening set for training the pharmacophore model. This is then screened against several pharmacophore queries, and the query that performs best is selected for further screening. In our case, for example, 60 very similar compounds would be generated for pharmacophores I and II, based on the MACCS fingerprints of the adamantane-based M2 inhibitors. Then, several pharmacophore queries including pharmacophore I and II are used to score the set of 60 similar compounds + 6 known compounds and identify which of the pharmacophore models best discriminates between the 6 known and the 60 highly-similar generated compounds.

Pharmacophore features that may hypothetically contribute to the activity of the M2(S31N) inhibitors reported in the literature were determined by how well the pharmacophore features of the hits fit into the generated common pharmacophores. This is expressed quantitatively as the root mean square deviation (RMSD), where compounds with the lowest RMSD are suggested as “hits.”

The virtual library for compounds from a subset of the marine and terrestrial natural product library [18] was generated using a previously explored approach for virtualizing the p-ANAPL library [19] and constituted 205 unique structures. From the computed parameters of the generated virtual library, 78 compounds showed no violations of Lipinski’s “Rule of Five” (34.1%) [54], while 140 compounds (68.3%) showed fewer than two violations.

### 4.3. Yeast Growth-Restoration Assays

Yeast growth-restoration assays were performed as described in Balgi and Roberge (2009) [28]. Briefly, *Saccharomyces cerevisiae* contain a multicopy plasmid for expression of M2(S31N) or M2(S31) obtained from the Udorn strain of influenza A virus under the control of a GAL1 promoter [29]. Cells were grown overnight at 37 °C and shaking at 200 rpm in Synthetic Complete minus leucine media (SC-L) at pH 6.5 plus 2% glucose to repress M2 expression [29]. Yeast cultures were then washed twice with sterile water and re-suspended in SC-L plus either 2% glucose or 2% galactose to an A620 optical density (OD) reading of 0.1. 100 µL of yeast cells re-suspended in SC-L plus galactose were then added to each well of a 96-well culture plate containing compounds at desired concentrations diluted in SC-L. A control 96-well plate was run in parallel containing *S. cerevisiae* in SC-L or SC-L plus 2% glucose or 2% galactose. Each experimental condition was performed at a minimum in triplicate. Following incubation at 37 °C in a humidifier box for 20 h, plates were gently vortexed at low speed for 1 min to re-suspend cells, and three OD readings at A620 were recorded using an Infinity M200 multimode plate reader (Tecan Life Sciences, Männedorf, Switzerland). The resulting data were then normalized to no inhibitor controls following background subtraction.

### 4.4. Preparation of Chebulagic Acid for Computational Docking Studies

Three-dimensional models of P1 and P2, which were based on expected hydrolysis reactions, were generated using the builder module of the graphical user interface of MOE software. Upon addition of partial charges, each part of the ligand was minimized in the gas phase using the OPLS-AA force field [55] until a gradient of 0.001 kcal/mol was attained. Interactions between P1 and P2 with M2 were modelled separately throughout the simulations.

### 4.5. Preparation of M2(S31N) for Computational Docking Studies

The solution nuclear magnetic resonance (NMR) structure of M2(S31N) complexed with M2WJ332 was retrieved from the Protein Data Bank (PDB ID: 2LY0) [20] and used as the starting point of all simulations. The ligand, all other small molecules, and solvent structures were then removed from the protein. This structure was re-examined and confirmed to contain no random or missing residues. Additional preparation of M2(S31N) for ligand docking was performed using MOE as follows: first, the structure was protonated using default parameters; second, partial charges were computed for each atom of the molecule; and third, energy minimization was performed with gas phase solvation parameters using the all atom OPLS-AA force field until a gradient of 0.001 kcal/mol was attained. The prepared ligand and protein files were then used as input files for subsequent docking calculations with MOE.

To generate the M2(S31) form, the structure described above was modified in situ to replace N31 with S31 as previously described [25] using the builder module of MOE. The structure was then relaxed 4.5 Å around the region where the modification took place. Docking validation was conducted with M2WJ332 (compound **4**) against the M2(S31N) form and amantadine against the M2(S31) form; both docked native ligands showed RMSD values < 1.5 Å with respect to the X-ray ligand structures. These results were used to locate the box size for conducting the docking of chebulagic acid.

### 4.6. Docking Calculations

P1 and P2 were separately docked towards the binding pocket of both M2(S31N) and M2(S31) around the vicinity of the co-crystalized M2WJ332 ligand using the Dock module implemented in MOE. To validate the docking protocol, the native ligand M2WJ332 was removed and re-docked into the M2 binding pocket in an attempt to reproduce the original ligand conformation within the binding pocket, with the lowest possible root-mean-square-deviation (RMSD) value. RMSD values at1.5 Å were defined as acceptable for docking validation of the native ligand back into the protein binding site. Several combinations of scoring functions for classifying the initially docked poses, followed by re-scoring of the force field parameterized refined poses, were performed, along with several attempts of validation. The best-retained combination was that of using the initial scoring functions which were set to “London dG” [56], with a force field parameterized refinement and a rescoring function set to “Affinity dG”, and the docked ligand giving a retained RMSD of 0.98 Å with respect to the native (co-crystallized) ligand. The London dG scoring function estimates the free energy of binding of the ligand to the protein from a given pose, and Affinity dG Scoring estimates the enthalpic contribution to the free energy of binding [57].

### 4.7. Generation of Amantadine-Sensitive and -Resistant Influenza Viruses

Recombinant influenza A viruses were generated using the reverse genetic system based on the A/Puerto Rico/8/34 (PR8) strain [30] provided by Dr. Y. Kawaoka (University of Wisconsin-Madison, Madison, WI, USA). Viruses were generated, as described previously [25]. Two PR8-derived recombinant influenza A viruses that differed only in M2 were used in this study: PR8_M2(S31N)_ carries M2 protein with V and N at positions 27, and 31, respectively, while PR8_M2(S31)_ carries V and S at positions 27, and 31, respectively.

### 4.8. Viral Cytopathic Assays

To test the inhibitory effect of compounds on virus replication, MDCK cell monolayers grown in 96-well plates were infected with 50× TCID_50_ of either PR8_M2(S31N)_ or PR8_M2(S31)_ in the presence of compound in DMEM+ containing 0.00075% Difco trypsin (BD Biosciences, San Jose, CA, USA) in quadruplicate, and as described previously [31]. Inhibitory effects were then scored for cytopathic effects, as observed by light microscopy, after 48–72 hours’ incubation at 37 °C. For each condition, the resulting inhibitory concentrations were determined from the results from at least three independent experiments.

Inhibitors’ effects were also tested by plaque reduction assays as described previously [25,58]. Briefly, approximately 100 plaque-forming units of either PR8_M2(S31N)_ or PR8_M2(S31)_ virus were mixed with test compounds and inoculated on confluent MDCK monolayers in six-well plates. After adsorption for 1 h, cells were washed twice with phosphate-buffered saline and overlaid with DMEM+ containing test compound at defined concentrations plus 0.00075% Difco trypsin and 1% SeaPlaque low melting agarose (Lonza, Richmond, BC, Canada), Following incubation at 37 °C for 3 days, cells were stained with 0.01% Neutral Red (Sigma, Oakville, ON, Canada) to visualize and count the plaques. For each condition, the resulting EC_50_ was determined from the results from at least three independent experiments.

## Figures and Tables

**Table 1:** Enthalpic contributions to binding free energy of chebulagic acid hydrolysis fragments to M2, along with interacting residues and M2 subunits.

Ligand	M2(S31N), Mutant	Interacting Residues in Mutant (M2 Subunits)	M2(S31), Wild Type	Interacting Residues in Wild Type (M2 Subunits)
P1	−39.75	His37 (B and D), Gly34 (B and D)	−11.78	Ala30 (A,B and D)
P2	−30.17	His37 (A and B)	+47.0	Ala30 (A)His37 (B, C and D)

**Table 2:** Effects of amantadine, M2WJ352, and chebulagic acid on viral replication, as measured by 50 * TCID_50_-based cytopathic assay. Results indicate mean ± SD of at least 3 independent experiments with at least 4 replicates per experiment.

Inhibitory Concentration Required vs. 50× TCID_50_ (µM)	PR8_M2(S31N)_	PR8_M2(S31)_
Amantadine (**1**)	>100	1.8 ± 2.3
M2WJ352 (**3**)	17.6 ± 19.3	>100
Agathisflavone (**10**)	>140	>140
Thiocillin I (**13**)	>65	>65
Chebulagic acid (**16**)	17.2 ± 15.2	32.4 ± 24.7

**Table 3:** Effects of amantadine, M2WJ352, and chebulagic acid on viral replication, as measured by plaque reduction assay. Results indicate mean ± s.e.m. from at least 3 independent experiments.

EC_50_(µM)	PR8_M2(S31N)_	PR8_M2(S31)_
Amantadine (**1**)	>5	0.16 ± 0.02
M2WJ352 (**3**)	3.2 ± 1.2	32.7 ± 16.1
Chebulagic acid (**16**)	60.9 ± 22.0	50.3 ± 26.4

## References

[1] Hayden F.G., De Jong M.D. (2011). Emerging influenza antiviral resistance threats. J. Infect. Dis..

[2] Nieva J.L., Madan V., Carrasco L. (2012). Viroporins: Structure and biological functions. Nat. Rev. Genet..

[3] Nieto-Torres J.L., Verdiá-Báguena C., Castaño-Rodriguez C., Aguilella V.M., Enjuanes L. (2015). Relevance of Viroporin Ion Channel Activity on Viral Replication and Pathogenesis. Viruses.

[4] Jalily P.H., Duncan M.C., Fedida D., Wang J., Tietjen I. (2020). Put a cork in it: Plugging the M2 viral ion channel to sink influenza. Antivir. Res..

[5] Sugrue R.J., Bahadur G., Zambon M.C., Hall-Smith M., Douglas A.R., Hay A.J. (1990). Specific structural alteration of the influenza haemagglutinin by amantadine. EMBO J..

[6] Alvarado-Facundo E., Gao Y., Ribas-Aparicio R.M., Jiménez-Alberto A., Weiss C.D., Wang W. (2014). Influenza Virus M2 Protein Ion Channel Activity Helps To Maintain Pandemic 2009 H1N1 Virus Hemagglutinin Fusion Competence during Transport to the Cell Surface. J. Virol..

[7] A Bright R., Medina M.-J., Xu X., Perez-Oronoz G., Wallis T.R., Davis X.M., Povinelli L., Cox N.J., I Klimov A. (2005). Incidence of adamantane resistance among influenza A (H3N2) viruses isolated worldwide from 1994 to 2005: A cause for concern. Lancet.

[8] Fiore A.E., Fry A., Shay D., Gubareva L., Bresee J.S., Uyeki T.M. (2011). Centers for Disease Control and Prevention (CDC). Antiviral agents for the treatment and chemoprophylaxis of influenza—Recommendations of the Advisory Committee on Immunization Practices (ACIP). MMWR. Recomm. Rep..

[9] Chaput L., Martinez-Sanz J., Saettel N., Mouawad L. (2016). Benchmark of four popular virtual screening programs: Construction of the active/decoy dataset remains a major determinant of measured performance. J. Chemin..

[10] Chen Y., Kops C.D.B., Kirchmair J. (2017). Data Resources for the Computer-Guided Discovery of Bioactive Natural Products. J. Chem. Inf. Model..

[11] Cichero E., D’Ursi P., Moscatelli M., Bruno O., Orro A., Rotolo C., Milanesi L., Fossa P. (2013). Homology Modeling, Docking Studies and Molecular Dynamic Simulations Using Graphical Processing Unit Architecture to Probe the Type-11 Phosphodiesterase Catalytic Site: A Computational Approach for the Rational Design of Selective Inhibitors. Chem. Boil. Drug Des..

[12] Franchini S., Battisti U.M., Prandi A., Tait A., Borsari C., Cichero E., Fossa P., Cilia A., Prezzavento O., Ronsisvalle S. (2016). Scouting new sigma receptor ligands: Synthesis, pharmacological evaluation and molecular modeling of 1,3-dioxolane-based structures and derivatives. Eur. J. Med. Chem..

[13] Tietjen I., Ntie-Kang F., Mwimanzi P., Onguéné P.A., Scull M.A., Idowu T., Ogundaini A., Meva’A L.M., Abegaz B.M., Rice C.M. (2015). Screening of the Pan-African Natural Product Library Identifies Ixoratannin A-2 and Boldine as Novel HIV-1 Inhibitors. PLoS ONE.

[14] Divsalar D.N., Simoben C.V., Schonhofer C., Richard K., Sippl W., Ntie-Kang F., Tietjen I. (2020). Novel Histone Deacetylase Inhibitors and HIV-1 Latency-Reversing Agents Identified by Large-Scale Virtual Screening. Front. Pharmacol..

[15] Naithani R., Huma L., Holland L., Shukla D., McCormick D., Mehta R., Moriarty R. (2008). Antiviral Activity of Phytochemicals: A Comprehensive Review. Mini Rev. Med. Chem..

[16] Andrae-Marobela K., Ghislain F.W., Okatch H., Majinda R.R. (2013). Polyphenols: A diverse class of multi-target anti-HIV-1 agents. Curr. Drug Metab..

[17] Tietjen I., Williams D.E., Read S., Kuang X.T., Mwimanzi P., Wilhelm E., Markle T., Kinloch N.N., Naphen C.N., Tenney K. (2018). Inhibition of NF-κB-dependent HIV-1 replication by the marine natural product bengamide A. Antivir. Res..

[18] Richard K., Williams D.E., De Silva E.D., Brockman M.A., Brumme Z.L., Andersen R.J., Tietjen I. (2018). Identification of Novel HIV-1 Latency-Reversing Agents from a Library of Marine Natural Products. Viruses.

[19] Ntie-Kang F., Onguéné P.A., Fotso G.W., Andrae-Marobela K., Bezabih M., Ndom J.C., Ngadjui B.T., Ogundaini A., Abegaz B.M., Meva’A L.M. (2014). Virtualizing the p-ANAPL Library: A Step towards Drug Discovery from African Medicinal Plants. PLoS ONE.

[20] Wang J., Wu Y., Ma C., Fiorin G., Wang J., Pinto L.H., Lamb R.A., Klein M.L., DeGrado W.F. (2013). Structure and inhibition of the drug-resistant S31N mutant of the M2 ion channel of influenza A virus. Proc. Natl. Acad. Sci. USA.

[21] Wang J., Ma C., Wang J., Jo H., Canturk B., Fiorin G., Pinto L.H., Lamb R.A., Klein M.L., DeGrado W.F. (2013). Discovery of Novel Dual Inhibitors of the Wild-Type and the Most Prevalent Drug-Resistant Mutant, S31N, of the M2 Proton Channel from Influenza A Virus. J. Med. Chem..

[22] Astrahan P., Flitman-Tene R., Bennett E.R., Krugliak M., Gilon C., Arkin I.T. (2011). Quantitative analysis of influenza M2 channel blockers. Biochim. Biophys. Acta (BBA) Biomembr..

[23] Alhadeff R., Assa D., Astrahan P., Krugliak M., Arkin I.T. (2014). Computational and experimental analysis of drug binding to the Influenza M2 channel. Biochim. Biophys. Acta (BBA) Biomembr..

[24] Zhao X., Jie Y., Rosenberg M.R., Wan J., Zeng S., Cui W., Xiao Y., Li Z., Tu Z., Casarotto M. (2012). Design and synthesis of pinanamine derivatives as anti-influenza A M2 ion channel inhibitors. Antivir. Res..

[25] Jalily P.H., Eldstrom J., Miller S., Kwan D.C., Tai S.S.-H., Chou D., Niikura M., Tietjen I., Fedida D. (2016). Mechanisms of Action of Novel Influenza A/M2 Viroporin Inhibitors Derived from Hexamethylene Amiloride. Mol. Pharmacol..

[26] Halgren T.A. (1996). Merck molecular force field. I. Basis, form, scope, parameterization, and performance of MMFF94. J. Comput. Chem..

[27] Daveu C., Bureau R., Baglin I., Prunier H., Lancelot J.-C., Rault S. (1999). Definition of a pharmacophore for partial agonists of serotonin 5-HT3 receptors. J. Chem. Inf. Comput. Sci..

[28] Balgi A.D., Roberge M. (2009). Screening for Chemical Inhibitors of Heterologous Proteins Expressed in Yeast Using a Simple Growth-Restoration Assay. Adv. Struct. Saf. Stud..

[29] Balgi A.D., Wang J., Cheng D.Y.H., Ma C., Pfeifer T.A., Shimizu Y., Anderson H.J., Pinto L.H., Lamb R.A., DeGrado W.F. (2013). Inhibitors of the Influenza A Virus M2 Proton Channel Discovered Using a High-Throughput Yeast Growth Restoration Assay. PLoS ONE.

[30] Neumann G., Watanabe T., Ito H., Goto H., Gao P., Hughes M., Perez D.R., Donis R., Hoffmann E., Hobom G. (1999). Generation of influenza A viruses entirely from cloned cDNAs. Proc. Natl. Acad. Sci. USA.

[31] Niikura M., Bance N., Mohan S., Pinto B.M. (2011). Replication inhibition activity of carbocycles related to oseltamivir on influenza A virus in vitro. Antivir. Res..

[32] Zhang X.-R., Kaunda J.S., Zhu H.-T., Wang D., Yang C.-R., Zhang Y.-J. (2019). The Genus Terminalia (Combretaceae): An Ethnopharmacological, Phytochemical and Pharmacological Review. Nat. Prod. Bioprospect..

[33] Li Y.-X., Yu S., Liu N., Proksch P., Lin W. (2012). Inhibitory effects of polyphenols toward HCV from the mangrove plant *Excoecaria agallocha* L.. Bioorg. Med. Chem. Lett..

[34] Vu T.T., Kim H., Tran V.K., Vu H.D., Hoang T.X., Han J.W., Choi Y.H., Jang K.S., Choi G.J., Kim J.-C. (2017). Antibacterial activity of tannins isolated from Sapiumbaccatum extract and use for control of tomato bacterial wilt. PLoS ONE.

[35] Pompermaier L., Marzocco S., Adesso S., Monizi M., Schwaiger S., Neinhuis C., Stuppner H., Lautenschläger T. (2018). Medicinal plants of northern Angola and their anti-inflammatory properties. J. Ethnopharmacol..

[36] Lee S.-I., Hyun P.-M., Kim S.-H., Kim K.-S., Lee S.-K., Kim B.-S., Maeng P.J., Lim J.-S. (2005). Suppression of the onset and progression of collagen-induced arthritis by chebulagic acid screened from a natural product library. Arthritis Rheum..

[37] Liu Y., Bao L., Xuan L., Song B., Lin L., Han H. (2015). Chebulagic acid inhibits the LPS-induced expression of TNF-α and IL-1β in endothelial cells by suppressing MAPK activation. Exp. Ther. Med..

[38] Shanmuganathan S., Angayarkanni N. (2018). Chebulagic acid Chebulinic acid and Gallic acid, the active principles of Triphala, inhibit TNFα induced pro-angiogenic and pro-inflammatory activities in retinal capillary endothelial cells by inhibiting p38, ERK and NFkB phosphorylation. Vasc. Pharmacol..

[39] Athira A.P., Helen A., Saja K., Reddanna P., Sudhakaran P.R. (2013). Inhibition of Angiogenesis In Vitro by Chebulagic Acid: A COX-LOX Dual Inhibitor. Int. J. Vasc. Med..

[40] Lu K., Basu S. (2015). The natural compound chebulagic acid inhibits vascular endothelial growth factor A mediated regulation of endothelial cell functions. Sci. Rep..

[41] Kashiwada Y., Nonaka G.-I., Nishioka I., Chang J.-J., Lee K.-H. (1992). Antitumor Agents, 129. Tannins and Related Compounds as Selective Cytotoxic Agents. J. Nat. Prod..

[42] Kumar N., Gangappa D., Gupta G., Karnati R. (2014). Chebulagic acid from Terminalia chebula causes G1 arrest, inhibits NFκB and induces apoptosis in retinoblastoma cells. BMC Complement. Altern. Med..

[43] Gao H., Huang Y.-N., Gao B., Kawabata J. (2008). Chebulagic Acid Is a Potent α-Glucosidase Inhibitor. Biosci. Biotechnol. Biochem..

[44] Huang Y.-N., Zhao D.-D., Gao B., Zhong K., Zhu R.-X., Zhang Y., Xie W.-J., Jia L.-R., Gao H. (2012). Anti-Hyperglycemic Effect of Chebulagic Acid from the Fruits of Terminalia chebula Retz. Int. J. Mol. Sci..

[45] Yang M.H., Vasquez Y., Ali Z., Khan I.A., Khan S.I. (2013). Constituents from Terminalia species increase PPARα and PPARγ levels and stimulate glucose uptake without enhancing adipocyte differentiation. J. Ethnopharmacol..

[46] Shyni G.L., Kavitha S., Indu S., Das Arya A., Anusree S.S., Vineetha V.P., Vandana S., Sundaresan A., Raghu K.G. (2014). Chebulagic acid from Terminalia chebula enhances insulin mediated glucose uptake in 3T3-L1 adipocytes via PPARγ signaling pathway. BioFactors.

[47] Takechi M., Tanaka Y., Takehara M., Nonaka G.-I., Nishioka I. (1985). Structure and antiherpetic activity among the Tannins. Phytochemistry.

[48] Lin L.-T., Chen T.-Y., Chung C.-Y., Noyce R.S., Grindley T.B., McCormick C., Lin T.-C., Wang G.-H., Lin C.-C., Richardson C.D. (2011). Hydrolyzable Tannins (Chebulagic Acid and Punicalagin) Target Viral Glycoprotein-Glycosaminoglycan Interactions To Inhibit Herpes Simplex Virus 1 Entry and Cell-to-Cell Spread. J. Virol..

[49] Lin L.-T., Chen T.-Y., Lin S.-C., Chung C.-Y., Lin T.-C., Wang G.-H., Anderson R., Lin C.-C., Richardson C.D. (2013). Broad-spectrum antiviral activity of chebulagic acid and punicalagin against viruses that use glycosaminoglycans for entry. BMC Microbiol..

[50] Kesharwani A., Polachira S.K., Nair R., Agarwal A., Mishra N.N., Gupta S.K. (2017). Anti-HSV-2 activity of Terminalia chebula Retz extract and its constituents, chebulagic and chebulinic acids. BMC Complement. Altern. Med..

[51] Lin C.-J., Liu C.-H., Jassey A., Lin C.-C., Li Y.-F., Richardson C.D., Lin L.-T. (2018). Small molecules targeting coxsackievirus A16 capsid inactivate viral particles and prevent viral binding. Emerg. Microbes Infect..

[52] Yang Y., Xiu J., Liu J., Zhang L., Li X., Xu Y., Qin C., Zhang L. (2013). Chebulagic Acid, a Hydrolyzable Tannin, Exhibited Antiviral Activity in Vitro and in Vivo against Human Enterovirus 71. Int. J. Mol. Sci..

[53] Li P., Du R., Wang Y., Hou X., Wang L., Zhao X., Zhan P., Liu X., Rong L., Cui Q. (2020). Identification of Chebulinic Acid and Chebulagic Acid as Novel Influenza Viral Neuraminidase Inhibitors. Front. Microbiol..

[54] Lipinski C.A., Lombardo F., Dominy B.W., Feeney P.J. (1997). Experimental and computational approaches to estimate solubility and permeability in drug discovery and development settings. Adv. Drug. Deliv. Rev..

[55] Jorgensen W.L., Tirado-Rives J. (1988). The OPLS [optimized potentials for liquid simulations] potential functions for proteins, energy minimizations for crystals of cyclic peptides and crambin. J. Am. Chem. Soc..

[56] Labute P. (2008). The generalized Born/volume integral implicit solvent model: Estimation of the free energy of hydration using London dispersion instead of atomic surface area. J. Comput. Chem..

[57] Lenzi O., Colotta V., Catarzi D., Varano F., Squarcialupi L., Filacchioni G., Varani K., Vincenzi F., Borea P.A., Ben D.D. (2011). Synthesis, structure–affinity relationships, and molecular modeling studies of novel pyrazolo[3,4-c]quinoline derivatives as adenosine receptor antagonists. Bioorg. Med. Chem..

[58] Song J.-M., Lee K.-H., Seong B.L. (2005). Antiviral effect of catechins in green tea on influenza virus. Antivir. Res..

